# The invisibility of men in South African violence prevention policy: national prioritization, male vulnerability, and framing prevention

**DOI:** 10.3402/gha.v8.27649

**Published:** 2015-07-29

**Authors:** Ashley van Niekerk, Susanne Tonsing, Mohamed Seedat, Roxanne Jacobs, Kopano Ratele, Roderick McClure

**Affiliations:** 1 Violence, Injury and Peace Research Unit, South African Medical Research Council – University of South Africa, Tygerberg, South Africa; 2 Institute for Social and Health Sciences, University of South Africa, Lenasia, South Africa; 3 Monash Injury Research Institute, Monash University, Melbourne, Australia

**Keywords:** violence, gender, vulnerable groups, prevention, policy, South Africa

## Abstract

**Background:**

South Africa has a significant violence problem. The exposure of girls and women to interpersonal violence is widespread, and the victimization of men, especially to severe and homicidal forms of aggression, is of considerable concern, with male homicide eight times the global rate. In the last two decades, there have been a plethora of South African policies to promote safety. However, indications suggest that the policy response to violence is not coherently formulated, comprehensive, or evenly implemented.

**Objective:**

This study examines selected South African national legislative instruments in terms of their framing and definition of violence and its typology, vulnerable populations, and prevention.

**Design:**

This study comprises a directed content analysis of selected legislative documents from South African ministries mandated to prevent violence and its consequences or tasked with the prevention of key contributors to violence. Documents were selected using an electronic keyword search method and analyzed independently by two researchers.

**Results:**

The legislative documents recognized the high levels of violence, confirmed the prioritization of selected vulnerable groups, especially women, children, disabled persons, and rural populations, and above all drew on criminological perspectives to emphasize tertiary prevention interventions. There is a policy focus on the protection and support of victims and the prosecution of perpetrators, but near absent recognition of men as victims.

**Conclusions:**

There is a need to broaden the policy framework from primarily criminological and prosecutorial perspectives to include public health contributions. It is likewise important to enlarge the conceptions of vulnerability to include men alongside other vulnerable groups. These measures are important for shaping and resourcing prevention decisions and strengthening primary prevention approaches to violence.

Globally, men have shorter lifespans and lose more years of their lives to death or disability than women do (1), with higher rates of heart disease, respiratory infections, cerebrovascular disease, HIV/AIDS, and road injuries (1, 2). Young men aged 25–39 years are especially affected, possibly because of the impact of injuries (3). South Africa is one of a number of countries plagued by high levels of injuries, which are driven by widespread, often gruesome violence (4–6). The country has a violence mortality rate of 65 per 100,000, nearly five times the global average (7), with nearly 2 million people annually seeking health care for violent injuries (8). Violence is endemic among women and girls, with a significant proportion of traumatic events linked to sexual violence, which is grossly underreported (9). Whereas the rate of female homicide involving intimate partners is six times the global rate (10), the exposure of men to violence – especially to severe and homicidal forms – is also disproportionate, with male homicide rates eight times the global average and the highest rates (184 per 100,000) reported for 15–29-year-olds (7).

Sexual violence toward boys and men is also notable: one study reported that 3–4% of young men are victims of rape (4), and another study highlighted its concentrations in prisons (11). Children are also vulnerable, at 5.5 homicides per 100,000 children younger than 18 years – about twice the global average. The homicide rate for boys, at 6.9 per 100,000, is nearly double that of girls, at 3.9 per 100,000. Gender differences increase with age, to reach a homicide rate among boys aged 15–17 years (21.7 per 100,000) nearly five times the rate among girls (4.6 per 100,000). Child abuse precedes nearly half of all child homicides in South Africa, with high levels of abuse underpinning vulnerability to such violence (12). There are many cases that involve sexual abuse, with 40% of girls and 17% of boys sexually abused (13). This extensive violence is compounded by episodes of high profile, state violence, such as that by the police on striking miners at Marikana in 2012 (14).

Violence is determined by a range of often inseparable dynamics located at individual, relationship, community, and societal levels (15). In South Africa, violence is marked by multiple social drivers, including widespread and racialized poverty, persistent unemployment, and extreme income inequality; patriarchal notions of masculinity that celebrate toughness and risk-taking; extensive exposure to abuse in childhood; access to firearms; excessive alcohol misuse; and weaknesses in law enforcement (4). Although there has been a steady overall decrease in violent deaths over the last decade, non-fatal violence to women has remained particularly high (4, 16). In view of the complex multifaceted nature of violence, prevention efforts are expected to include multisectoral and multidisciplinary contributions and legislative mechanisms to enable a socially cohesive response (15, 17, 18).

The South African government has prioritized the prevention of violence through a range of intersectoral initiatives. The recently formulated National Development Plan (NDP) aims to reduce violence by half by 2030 (19) with collaborative prevention efforts involving the health, justice, and police sectors through national government stewardship. These efforts have tended to focus on criminal justice measures and services for victims rather than on primary prevention, with gender appearing to feature significantly in the existing secondary and tertiary prevention responses (4, 20). The South African government response is aligned to the resolutions made at the Sixty-Seventh World Health Assembly (21), with a focus on strengthening health systems to address violence, especially against women, girls, and children. There is recognition that boys and young men are amongst those most affected by violence; however, the Assembly primarily emphasizes interventions for women, girls, and children. Their purpose is primarily to reduce child maltreatment and substance and alcohol abuse and to provide health and social care and/or rehabilitation for victims, but also for perpetrators. The South African response would appear aligned to this limited inclusion of support to boys (as children) in vulnerable settings, and men as perpetrators.

Despite this global focus on women and children as the most important victims of violence, there is emerging recognition of men's vulnerability to violence, especially as perpetrated by male strangers or acquaintances, with pockets of recent research on male violent victimization, including sexual victimization (22), violence during conflict and post-conflict situations (23), and, more controversially, male intimate partner violence victimization (e.g. 24, 25). Despite this, the international conventions and country plans seem to have maintained a focus on policies and programs aligned to the Sixty-Seventh World Health Assembly (21). This study examines the definition and delineation of violence and its typology, vulnerable populations, and prevention in selected South African national legislative instruments.

## Methods

The focus is on key legislative documents that deal with violence prevention. The study draws on a number of core concepts that define violence prevention and policy, respectively (see Box 1).

***Box 1*. Terminology**

The terminology used as a basis for the study closely follows that used by the WHO, by government or by agencies on behalf of government. Violence is defined in the *World Report on Violence and Health* as follows:

… the intentional use of physical force or power, threatened or actual, against oneself, another person, or against a group or community that either results in or has a high likelihood of resulting in injury, death, psychological harm, maldevelopment, or deprivation. (26: p. 5)

Violence can be further classified as self-inflicted (i.e. suicide or self-mutilation), interpersonal (i.e. homicide, intimate partner, sexual abuse, child abuse and neglect, elder abuse), and collective (i.e. political violence, acts of terrorism, and gang violence) (18). In this article the violence is restricted to interpersonal and collective forms, as the more prominent types currently in South Africa (4).

The WHO states that policy can be defined in a number of different ways, with the following definition of violence prevention policy:

A policy on violence and injury prevention is a document that sets out the main principles and defines goals, objectives, prioritized actions and coordination mechanisms, for preventing intentional and unintentional injuries and reducing their health consequences. (18: p. 5)

### Source documents

This study uses public health conceptualizations of violence prevention policy (15, 18). For purposes of the initial keyword search, documents searched included white papers, green papers, draft bills, bills, acts, notices of or official amendments to acts (including regulations, proclamations, promulgations, and national instructions), and other official documents such as policy guidelines that provide implementation instructions for policy development or implementation (see Box 2).

***Box 2*. The legislative process in South Africa**

**Green paper**

A *green paper* is a discussion document regarding a possible new policy that is needed. The green paper is used to elicit responses from the public on a policy issue via publication in the *Government Gazette*, and once responses have been received and discussed it usually becomes a white paper (27).

**White paper**

A *white paper* is a policy drafted by a government department on a particular issue that has been published for public comment. It can include legislative and administrative proposals for government implementation. It is the final policy plan (27).

**Bill**

A *bill* is a draft of a new act or law that has been tabled in Parliament but not yet passed. Once it has been passed, it becomes a new law. An amendment bill drafts proposed changes to an existing act (27, 28).

**Act**

Once a bill has been passed by Parliament and has been signed by the president, it becomes an act (or law) and is published in the *Government Gazette* (27, 28).

**Amendment to an act/amendment act**

An *amendment act* (or amendment to an act) is a proposed change or addition to an existing act or bill that is eventually accepted and becomes part of one or more acts. As it will eventually be part of an act, it is just as important a policy document as the act itself (27).

**Regulations/instructions**

Regulations are the official rules for the implementation of a law or how the aims of a law are to be carried out. The administrative agencies within government implement these regulations to achieve the legislative intent of that particular act. A national instruction is similar to the regulation (27).

**Legislation**

*Legislation* is an overarching term that refers to laws, statutes, and acts. The legislative process refers to the steps taken within Parliament to bring about a new law (28).

### Search strategy

The search involved three steps: 1) identification of departments holding a violence prevention mandate, 2) sourcing and selection of violence-related policies and legislation, and 3) the finalization of the document pool by keywords and accessibility. Ten national government departments were identified as comprising violence prevention mandates: the Department of Basic Education, Department of Correctional Services, Department of Health, Department of Human Settlements, Department of Justice and Constitutional Development, Department of Social Development, Department of Sport and Recreation, Department of Transport, Department of Trade and Industry, and the Police Service. We selected policy and legislative documents published or amended since 1994 by looking closely at document titles and executive summaries. We included any document that appeared at an initial scan to address issues of violence, injury, or safety. This method of selection is not exhaustive, as relevant documents may have been missed or may not have been available on the government website. The documents that were included were sourced over February to May 2012.

We electronically searched each selected document for the following keywords: violence, injury, and safety. The keyword *prevention* was initially included in the search, but was discarded after it was found that the term was too broad and included too many other unrelated topics. It was decided that *prevention* as a relevant concept would be identified during the qualitative content analysis, when its relation to one of the other keywords could be verified. *Injury* was included as a common indicator of violence and violence prevention research. Limitations to the electronic keyword searches included the inferior quality of some documents, whereas others were in some respects electronically corrupt, making searches unreliable. Documents were grouped together into four categories: 1) acts/amendment acts (including legislation/law, promulgations), 2) policy and developing legislation (green/white papers, bills, policies, amendment bills), 3) internal documents (annual reports, strategic plans/frameworks, implementation/action plans), and 4) legislative instruments (guidelines to acts, policy guidelines, instructions, regulations).

### Final document pool

Of the selected documents, 90% (115 documents) were included in the initial document pool for the keyword searches. The distribution of documents was relatively even: acts and amendments, 30%; policy and developing legislation, 19%; internal documents, 26%; and legislative instruments, 25%. Of these, 108 documents were electronically searchable. The total number of keyword hits for these documents was 921 for the word *violence*, 1,892 for *safety*, and 322 for *injury*. The final document pool focused only on acts/amendment acts, as well as policy and developing legislation (53 documents), and excluded others. These types of documents were considered most relevant to the study, in that acts/amendment acts are the highest ranking forms of policy, that is, enforceable law. By contrast, policy and developing legislation such as green papers, white papers, and bills are the document types through which emerging legislation is finalized by government, with the input of the public at various stages. This pool was then reduced by selecting only those documents with at least eight keyword hits for any one (or more) of the keywords, in order to focus on those documents with the greatest likelihood of yielding relevant information. The final document pool included 20 documents (see Box 3).

**Box 3:** Final legislation and developing legislation document pool

Ministry	Name	Reference
DOH	National Youth Policy	South Africa. Parliament. National Youth Commission. 2009. *National youth policy 2009-2014* [Online]. Available from: www.info.gov.za/view/DownloadFileAction?id=102384 [cited 9 September 2013].
DOJ&CD	Child Justice Act	*Child Justice Act (No. 75 of 2008)* [Online]. Available from: www.info.gov.za/view/DynamicAction?pageid=623&myID=194132 [cited 9 September 2013].
DOT	National Scholar Transport Policy	South Africa. Department of Transport. 2009. *Final draft national scholar transport policy* [Online]. Available from: www.fedsas.org.za/downloads/10_52_24_National Scholar Transport Policy.pdf [cited 9 September 2013].
DSD	Child Care Act	No longer in use.
DSD	Children's Act	*Children's Act (No. 38 of 2005)* [Online]. Available from: www.info.gov.za/view/DownloadFileAction?id=67892 [cited 9 September 2013].
DSD	Draft green paper on families	South Africa. Department of Social Development. 2011. *Green paper on families: promoting family life and strengthening families in South Africa* [Online]. Available from: www.info.gov.za/view/DownloadFileAction?id=152939 [cited 9 September 2013].
SAPS	Firearms Control Act	*Firearms Control Act (No. 60 of 2000)* [Online]. Available from: www.info.gov.za/view/DownloadFileAction?id=68229 [Accessed 9 September 2013].
SAPS	Sexual Offences Amendment Act	*Criminal law (sexual offences and related matters Act (No. 32 of 2007))* [Online]. Available from: www.saps.gov.za/docs_publs/legislation/sexual_offences/sexual_offences_act32_2007_eng.pdf [cited 9 September 2013].
SAPS	White Paper on Safety and Security	South Africa. South African Police Service. 1998. *White paper on safety and security* [Online]. Available from: www.info.gov.za/whitepapers/1998/safety.htm [cited 9 September 2013].
DCS	White Paper 8 on Corrections in South Africa	South Africa. Department of Correctional Services. 2005. *White paper on corrections in South Africa (8)* [Online]. Available from: www.info.gov.za/view/DownloadFileAction?id=68870 [cited 9 September 2013].
DOJ&CD	Criminal Procedure Act	*Criminal Procedure Act (No. 51 of 1977)* [Online]. Available from: www.justice.gov.za/legislation/acts/1977-051.pdf [cited 9 September 2013].
DOJ&CD	Domestic Violence Act	*Domestic Violence Act (No. 116 of 1998)* [Online]. Available from: www.info.gov.za/view/DownloadFileAction?id=70651 [cited 9 September 2013].
DOJ&CD	Prevention of Public Violence and Intimidation Act	*Prevention of Public Violence and Intimidation Act (No. 139 of 1991)* [Online]. Available from: www.justice.gov.za/legislation/acts/1991-139.pdf [cited 9 September].
DOJ&CD	Prevention and Combating of Trafficking in Persons Bill	South Africa. Department of Justice and Constitutional Development .2009. *Prevention and Combating of Trafficking in Persons Bill* [Online]. Available from: www.justice.gov.za/legislation/invitations/20090508_EnglishTIP.pdf [cited 9 September 2013].
DOJ&CD	Protection from Harassment Bill	South Africa. Parliament. 2010. *Protection from Harassment Bill* (B 1B 2010) [Online]. Available from: www.info.gov.za/view/DownloadFileAction?id=147773 [cited 9 September 2013].
DOT	Draft National Non-Motorised Transport Policy	South Africa. Department of Transport. 2008. *Draft national non-motorised transport policy* [Online]. Available from: www.joburg-archive.co.za/2009/pdfs/transport/nmt_policy.pdf [cited 9 September 2013].
DOT	White Paper on National Transport Policy	South Africa. Department of Transport. 1996. *White paper on national transport policy*. [Online]. Available from: www.info.gov.za/whitepapers/1996/transportpolicy.htm [cited 9 September 2013].
DSD	Children's Amendment Bill	South Africa. Department of Social Development. 2006. *Children's Amendment Bill (B 19B)* [Online]. Available from: www.info.gov.za/view/DownloadFileAction?id=65470 [cited 9 September 2013].
SAPS	Protection of Constitutional Democracy against Terrorist and Related Activities Act	*Protection of Constitutional Democracy against Terrorist and Related Activities Act (No. 33 of 2004)* [Online]. Available from: www.info.gov.za/view/DownloadFileAction?id=67972 [cited 9 September 2013].
DOH	White Paper for the Transformation of the Health System of South Africa	South Africa. Department of Health. 1997. *White paper for the transformation of the health system of South Africa* [Online]. Available from: www.info.gov.za/view/DownloadFileAction?id=187656 [cited 9 September 2013].

*Note*: DOH, Department of Health; DOJ&CD, Department of Justice and Constitutional Development; DOT, Department of Transport; DSD, Department of Social Development; SAPS, South African Police Service; DCS, Department of Correctional Services.

The 20 documents used in this study were therefore authorized by the South African Parliament, the mandated legislative authority in the country's national sphere of government. Parliament passes new laws, amends existing laws, and repeals old laws. There are also provincial legislatures with this authority in the provincial sphere of government, but only with respect to provincial laws, and, similarly, local municipal councils have this authority with respect to municipal by-laws. The powers of the provincial governments are limited to listed ‘functional areas’; in some instances these are shared with those of the national government, whereas in other areas the provincial governments have exclusive powers. These functional areas include economic development, education, environmental affairs, finance, health, human settlements, police or public safety, public works, roads and transport, social development, and sport and recreation, with many of these responsibilities involved in the implementation of legislation and policy relevant for the implementation of violence prevention activities (29).

### Directed content analysis

The documents were analyzed deductively, using directed content analysis, comprising the examination of documents and identification of relevant themes (30, 31). Directed content analysis is guided by a structured process that differs from conventional approaches to content analysis, using existing theory or prior research to identify key concepts or variables as initial coding categories (31). Two researchers independently highlighted document extracts related to key descriptions of violence and its prevention. A third researcher performed spot-checks to ensure the consistency of themes and alignment with the study aims. The findings were summarized with illustrations drawn from selected documents (30–32).

## Findings and discussion

### National prioritization of violence and its typology

Most of the selected legislative documents recognized violence as a national priority, focusing on specific violence-related behavior and emphasizing enforcement. Government's Programme of Action, the Presidency's 12 Key Outcomes, NDP, and several ministries all emphasize the high levels of violence and call for the reduction of risk factors (19). For instance, South Africa's violence problem is even viewed as an obstacle to the mainstreaming of alternative modes of transport, including walking and cycling. The Draft National Non-Motorised Transport Policy links its transport mandate to violence prevention:

Street lights invite more and more people to walk because they indicate safety, and to the extent that they are always lit […] they provide necessary levels of surveillance and pedestrians being seen by others who can help in the event of difficulties and insecurity. (33: p. 38)

Despite this prioritization, the current analysis revealed a number of limitations in the selected legislative documents. First, documents often lacked clear definitions of violence prevention terms. Many documents used the terms *violence* and *injury*, or *crime* and other terms from criminological typologies, yet these were not explicitly defined. The final draft of the National Scholar Transport Policy, for example, extensively refers to injuries, hijacking, violence, safety, and security, but without explicit definitions (34). *Safety* and *security* were used in several documents, with *safety* tending to be associated with unintentional or *accidental* injury and *security* linked to intentional injury arising from violence. Although the documents make some reference to violence, with the accent on interpersonal and criminal-related violence, the use of the terms do not always resonate with international conventions (15, 18, 35). Second, the analyzed documents do not appear to recognize the full typology of violence evident in South Africa, with no differentiation of certain disaggregated forms of violence. For instance, within interpersonal categories there was typically no distinction between stranger and intimate partner violence; within collective violence, there was no distinction between gang as opposed to community-organized violence (26). Such typological differentiations are clearly described in prevention-oriented public health and are considered important for shaping prevention decisions, for example, around the current manifestations of community ‘xenophobic’ violence (17).

Third, the existing typological definitions tended to only refer to criminological and legal conceptions. International illustrations indicate that comprehensive definitions are required to guide effective violence prevention (15, 17). Countries such as Australia and Canada have been noted as examples of countries with successful injury-prevention strategies with inclusive, uniform, and clear descriptions of injury and violence-related concepts in its legislation (18). The Canadian Injury Prevention Strategy, for example, uses both public health and legal frameworks for its concepts and incorporates evidence-based practices, while cautioning against the use of vague terminology that may inadvertently misdirect injury prevention responses (18, 35).

### Vulnerability: children, women, disability, and rurality

Fourth, the analyzed documents offered limited consideration of men as a vulnerable group. The documents revealed a clear emphasis on the vulnerability of selected groups, especially women and children and particularly black women and children, as those most affected by violence, while also highlighting the overrepresentation of vulnerability amongst poor and rural populations and their associated needs for care and development. Within the context of the South African criminal justice system, black children have been recognized as a specific vulnerable group, with children and youth identified as significant beneficiaries of state interventions (36). For example, children and youth in conflict with the law are earmarked for special care:

The Constitution, while envisaging the limitation of fundamental rights in certain circumstances, emphasizes the best interests of children, and singles them out for special protection, affording children in conflict with the law specific safeguards. (36: p. 2)

The Children's Act also consistently makes special reference to children with disabilities:

In any matter concerning a child with a disability due consideration must be (a) providing given to the child with parental care, family care or special care as and when appropriate; (b) making it possible for the child to participate in social, cultural, religious and educational activities, recognizing the special needs that the child may have; (c) providing the child with conditions that ensure dignity, promote self-reliance and facilitate active participation in the community; and (d) providing the child and the child's care-giver with the necessary support services. (37: p. 41)

The Children's Act identifies women and children as being at higher risk for violence and the related social and economic harm than any other population, prioritizing the alleviation of factors that increase these groups’ vulnerability:

State Parties shall take or strengthen measures, including through bilateral or multilateral cooperation, to alleviate the factors that make persons, especially women and children, vulnerable to trafficking, such as poverty, underdevelopment and lack of equal opportunity. (37: p. 135)

and especially through the recognition of gender inequality:

Gender inequality (inequality in terms of the power relations between men and women), in terms of popular attitudes and beliefs in favor of male domination, and the inadequate service offered by the criminal justice system to women, contributes to the high levels of violence perpetrated against women. (38: p. 51)

This focus is consistent with the national governmental and non-governmental drive to draw public attention to and strengthen the organized collective responses to violence against women and children (4, 12). However, what is not included is the vulnerability of men.

### Invisibility of men

Men are overrepresented in South African reports of homicide and other severe forms of interpersonal violence (4–6), reflected in global homicide patterns (39), but also in other indicators of vulnerability, such as the United Nations Office on Drugs and Crime (UNODC) Global Report on Trafficking, which indicates for example that 25% of global trafficking victims are men trafficked for purposes of labor (40). Despite these reports, the South African legislative documents in this study leave an impression that men are unaffected by violence and related human rights or social violations such as trafficking. Whereas the care of women and children as the victims of violence has been fittingly identified, the South African legislative response has tended to ignore this overrepresentation of males as victims (4). For instance, the White Paper on Safety and Security (41) makes no mention of men as vulnerable to victimization, despite current evidence (42–44). The recognition of male police officers as frequent victims of violent crime is an exception:

Many police officers have become victims of violent crimes. It must be acknowledged that police officers in South Africa have a much greater chance of being victimized by violence than do citizens. However, some of us have lost sight of the commitment and huge sacrifices being made by thousands of policemen and women. (41: p. 4)

Our analysis furthermore revealed a tendency to omit or limit references to the gender of perpetrators or offenders. In most of the documents, offenders are genderless, except where identified as being violent toward women, in which case their maleness is often also implied. Men's violence, through either perpetration or victimization, appears not to be considered as a gendered phenomenon. In a small selection of instances, however, the gender of offenders is recognized. For example, chapter seven of the White Paper on Corrections in South Africa is dedicated to the question ‘Who are South Africa's offenders?’ and calls for the entrenchment of the legislative rights of all offenders. However, it goes further to single out the rights of women and child victims of male offenders, emphasizing only the correctional sentence plans for the latter, while silent on the rights of the vast numbers of male victims and the need for correctional plans for those male offenders whose victims are not women or children, but other males:

The issue of Gender will be a crucial element in these Correctional Sentence Plans, particularly in relation to male offenders whose victims were women and children. (45: p. 13)

The limited engagement with evidence of the vulnerability of men to violence appears to undermine the consideration of boys and men as legitimate recipients of violence prevention interventions, echoing concerns that black males have been inadvertently pathologized in South Africa (46). There are, however, exceptions; for example, men are considered essential to South African families, with the absence of fathers creating a role model void for male youth, which in turn contributes to delinquency and violence (44). The White Paper on Corrections discusses the family unit as protective; however, even here, the focus is on absent mothers and less on absent fathers, largely included as part of ‘both parents’, rather than explicitly as ‘fathers’ or ‘men’:

Families living on the edge of survival have a great possibility of becoming dysfunctional. Many children must grow up in families without a mother, or even both parents. Factors such as poverty, the migrant labor system, outdated traditions, the effects of AIDS and the changing roles of men and women, cause hunger, hardship and challenges to traditional socialization processes. (45: p. 34)

### Framing prevention

Finally, many of the documents focus only on victim care and support and, by implication, consider violence prevention only as a secondary or tertiary prevention activity directed at minimizing the impact of violence. The Criminal Law (Sexual Offences and Related Matters) Amendment Act 2007, for example, focuses on services aimed at eliminating secondary traumatization to victims:

… providing certain services to certain victims of sexual offences, *inter alia*, to minimise or, as far as possible, eliminate secondary traumatization, including affording a victim of certain sexual offences the right to require that the alleged perpetrator be tested for his or her HIV status and the right to receive Post Exposure Prophylaxis in certain circumstances. (38: p. 4)

The Child Justice Act 2008 also emphasizes rehabilitation and restoration through:

… recognis[ing] the present realities of crime in the country and the need to be proactive in crime prevention by placing increased emphasis on the effective rehabilitation and reintegration of children in order to minimize the potential for re-offending. (36: p. 3)

Prevention was predominantly approached with a reactive or rehabilitative, often enforcement-based approach to violence. Prevention is, in other instances, described in broader and less coherent terms, often without specific reference to violence, and referring instead only to disease or illness, for example, “‘prevention’ means ensuring that diseases or illnesses do not occur” (47: p. 186), but with no further clarification of the levels of prevention (15, 17, 26).

In other instances, our analysis identified some consideration of primary prevention. The National Youth Policy identified the stimulation of recreational activities to foster social cohesion as a protective factor:

There is a need to engage youth in recreational activities that enhance social cohesion. This includes development of community-based infrastructure promoting arts, culture, sport and the overall entertainment of youth. The need for such facilities, which should offer a range of activities, far outweighs the supply. (48: p. 27)

A similar marginal focus was reflected in the White Paper on Safety and Security, which emphasized the following:

… enhancing social crime prevention activities to reduce the occurrence of crime. This requires … focusing on issues relating to the role of the police within the constitutional order, their legitimacy and the delivery of an effective service to the public. On the other hand, this also requires a dedicated focus on preventing citizens from becoming victims of crime. (14: p. 4)

Our analysis resonates with other reports of the South African state's focus on reactive methods, with the national response to violence characterized by criminal justice enforcement (4, 14), with national plans for the prevention of intimate partner violence, child maltreatment, and armed violence. There are no national action plans for youth and gang violence (49), but there are some policies and program activities that support youth at high risk to violence (e.g. through support of secondary schooling), a range of violence prevention programs (from preschool enrichment, social development or life skills training, mentoring, and after-school programs), and diversion interventions for youth at risk (49). The South African response echoes the resolutions of the Sixty-Seventh World Health Assembly (21) and the *Global Status Report on Violence Prevention* (50). Globally, two-thirds of countries report national action plans to address child maltreatment and violence against women, but about half report plans for youth violence prevention and just 40% report plans for gang violence prevention, reflecting the more limited attention to the violence burden faced by boys and men (50).

Although South Africa has greatly invested in women's and girls’ empowerment and gender equality, boys and especially men are still considered primarily as perpetrators of violence against women and girls. However, boys and men are themselves vulnerable to extreme physical and sexual violence and health problems, with the significant male violence burden suggestive of a widening of conception of gender and renewed understanding of the gender-violence nexus (4–7, 16). However, calls to broaden the understanding of the nexus between gender and violence need to be disentangled from the politics of gender relations and concerns that a male violence focus may diminish the hard-earned achievements obtained through feminist scholarship (16). This study recommends the strengthening of the South African focus on male victimization support and perpetration prevention, through the development of a national action plan and provision of a national focal point for this plan. Such a plan should strengthen the multisectoral basis for male violence prevention that involves the public and private sectors (for example, health, education, criminal justice, social services, and business) and civil society, that adopts a multisectoral approach, and that has high level leadership to coordinate collaborative activities. There are currently multiple agencies that take responsibility for violence prevention activities in South Africa, through a governmental cluster system and Inter-Ministerial Committees, but no single coordinating authority (49).

## Conclusions

Despite the complexities of violence prevention and the global call for multisectoral national action plans (18), the South African legislative responses, while recognizing violence as a national priority, are in crucial aspects partial and limited in scope. These responses have rightfully placed the accent on women, children, and sometimes the disabled or rural residents as victims, but they have tended to neglect the implications arising from the overconcentration of males in violent victimization and perpetration.

Since the 1990s, there has been increasing and substantial support for the socio-economic rights of children and women, with significant efforts to promote these groups’ quality of life through legislation that focuses on violent offences, such as domestic violence, trafficking, and sexual offenses. These efforts and the consequent legislation, however, do not consider the widespread vulnerability of men especially to severe forms of violence. Instead, these policy and legislative instruments are largely reactive, drawing on criminological perspectives and therefore focusing on tertiary prevention, which includes preventing reoffending, protecting victims, and correcting wrongful behavior. Recently, there has been a notable international shift toward primary prevention (18). In South Africa, legislative efforts are only just emerging to redress this oversight and consider multisectoral approaches that prioritize primary prevention alongside traditional secondary and tertiary approaches, but these efforts are essential to further the country's violence prevention commitments.

The neglect of male victims and limited consideration of offenders signals a failure in policy conceptions of vulnerability. In South Africa, as elsewhere, there are significant barriers to effective research to policy translation, including inabilities to access relevant research, the representation or packaging of such research, and the influence and priorities of interest groups (51, 52). This study suggests strengthened national policy, legislative, and social approaches to gender- and intimate partner–based violence prevention that include consideration of boys and men (as victims or perpetrators), through greater engagement and collaboration between the government, society, and science clusters, with greater utilization of existing evidence in the formulation of policy. Policy development models suggest that different types of communication strategies may be required, which, in the case of the interaction within and between the science and policy clusters, may also imply improvements in existing relations between the actors involved (52).
